# Sambucus in an Unusual Case of Sweating

**Published:** 1892-03

**Authors:** J. C. White

**Affiliations:** Portchester, N. Y.


### SAMBUCUS IN AN UNUSUAL CASE OF SWEATING.

J. C. White, M. D., Portchester, N. Y.

Miss E. D. M., age eighteen, light complexion, blue eyes, dark hair and of slight figure.

Menstruated at about fifteen, since which time had periods of copious sweating. The quantity seems incredible. From two to five quarts of water would literally pour out of every pore in the body in the space of one minute of time! There would be no premonitions except a little moisture of the face and hands. She kept a wash-tub in a pantry close to her bedside, and at the first indication of the sweating would jump into the tub, often leaving a trail of the perspired water on the carpet. I had frequently heard of this case, but must confess that I was incredulous until I actually saw it, and to see was to be convinced. The sweating always occurred during her waking hours; was never roused from sleep by it. It occurred more frequently at the time of her menstruation—occasionally twice daily—usually from one to three days apart. Often during the ten or fourteen days preceding menses would have none.

She was very pale. Flesh soft and cold to touch. During the sweating periods—*i. e.*, when they occurred from one to three days apart—she suffered much pain, the only intermission being from six to nine P. M. The pains were sharp, shooting, and cutting, mostly in stomach, abdomen, head, and upper limbs, always coming on gradually and leaving abruptly. The latter characteristic of pain being so marked that after being confined to the bed for days or weeks would jump up and dress, declaring that she was well. She was always bright, cheerful, and seemed less conscious of pain when entertained by her friends.

Her menses were regular as to time—flow scanty and of dark color.

I was called to see this case in March, 1891. I confess that I had not read the text of Sambucus very carefully. I expected every case of Sambucus to "wake out of sleep with the sweat." As an "old school" man, my "old tricks" naturally recurred to me. I had produced copious sweat with Jaborandi. I procured some and gave it from 30C to 40M potency without any appreciable result.

I then gave her a dose of Sepia$^{cc}$. She expressed herself much relieved, went over her next monthly period without sweat, but returned before the next one. Taking the character of pain as the guide I then gave Sul. acid$^{1m}$. She expressed herself much relieved of the pain. The sweat was somewhat lessened, but as a homœopathic physician it was unsatisfactory to me.

During the month of September she was confined to the bed and had a profuse sweat as often as every second day, besides many minor ones. I then gave Sambucus-niger$^{cc}$ two doses. The effect was magical, as the correct remedy always is, for there has been no sweat since. About six weeks after she complained of the characteristic pain in stomach and bowels. I gave a dose of Samb.$^{500}$. That is all the medicine she has had. She has gradually improved and looks now the picture of health. Why did I not give this remedy at first? She had been attended by several homœopathic physicians, covering a period of three years, before I saw her. By one of acknowledged eminence from New York City. Why did they not give it?

